# Effects of different dietary metabolizable energy levels on growth performance, liver metabolism, and gut microbiota in growing broilers

**DOI:** 10.1016/j.psj.2026.107520

**Published:** 2026-07-28

**Authors:** Xingyue Wu, Leilei Wang, Yuelong Chen, Yanqun Huang, Huaiyong Zhang, Rui Zheng, Siqiang Liu, Zike Feng, Wen Chen, Xuemeng Si

**Affiliations:** a Department of Companion Animal Science, Henan Agricultural University, Zhengzhou 450046, China; b Anyang Bonongli Biotechnology Company Limited, Anyang, 455000, China

**Keywords:** Broiler, Metabolizable energy, Growth performance, Antioxidant capacity, Gut microbiota

## Abstract

This study investigated how graded dietary metabolizable energy (ME) levels affect growth performance, and metabolic regulation in broilers, with particular focus on oxidative status, hepatic metabolism, and gut microbiota. A total of 480 one-day-old male AA broilers were reared under standard conditions until 21 days of age, after which they were assigned to four dietary treatments containing 3050, 3150, 3250, or 3350 kcal/kg. Growth performance was not significantly affected by dietary ME, as body weight, average daily gain, average daily feed intake, and feed conversion ratio were similar among treatments (*P_ANOVA_* > 0.05). However, dietary ME significantly influenced relative gizzard weight and immune organ indices, including the thymus, spleen, and bursa of Fabricius (*P_ANOVA_* < 0.05). Higher dietary ME enhanced antioxidant capacity, reflected by increased serum SOD and GSH-Px activities, while TNF-α decreased with increasing ME (*P_ANOVA_* < 0.05). Liver expression of genes involved in glucose metabolism, lipid metabolism, and insulin signaling was modulated by dietary ME. In the ileum, FABP6 expression increased with increasing dietary ME (*P_ANOVA_* < 0.05), whereas FBP1 and INSR expression decreased (*P_ANOVA_* < 0.05). Collectively, these findings suggest that moderate dietary ME levels may support metabolic stability and antioxidant capacity without affecting growth performance.

## Introduction

Dietary energy supply is one of the major determinants of feed cost and nutrient utilization efficiency in broiler production. Therefore, scientifically optimizing dietary energy levels is critical for reducing feed consumption per unit of animal product and lowering overall production costs (Abdulla et al., 2019; Massuquetto et al., 2020). Metabolizable energy (ME) is commonly used as the basis for formulating poultry diets because it directly influences feed intake, growth, and feed conversion efficiency. Traditionally, broilers are considered capable of adjusting voluntary feed intake in response to dietary energy density to maintain relatively stable energy intake (Ceylan et al., 2023; Ge et al., 2019; Wang et al., 2025). Therefore, increasing dietary ME within an appropriate range is expected to reduce feed intake and improve feed conversion without compromising growth performance. However, this energy-driven intake regulation may vary with genotype, age, nutrient balance, and feeding conditions (Jia et al., 2024).

In practical settings, feed intake does not always decline proportionally when dietary energy exceeds a certain level (Zhao and Kim, 2017), indicating that intake regulation may be influenced by metabolic feedback mechanisms, nutrient balance, or gastrointestinal capacity constraints (Noblet et al., 2024; Toghyani et al., 2025). Studies have shown that energy intake is closely linked to metabolic regulation, oxidative status, and immune function, particularly through the central role of the liver in coordinating lipid and glucose metabolism (Szczepańska and Gietka-Czernel, 2022; Tian et al., 2025). Additionally, the gut microbiota, as an important mediator of nutrient utilization and host metabolism, may also respond to variation in dietary energy, potentially influencing host physiological homeostasis. However, an integrated evaluation of how graded dietary energy levels affect metabolic regulation, antioxidant capacity, and gut microbiota in broilers remains limited.

Dietary energy level influences metabolic partitioning and overall physiological status, and is a key determinant of nutrient utilization efficiency and metabolic homeostasis in broilers (Diehl et al., 2024). The liver, as the primary site of lipogenesis in poultry, is the central organ responsible for sensing and responding to dietary energy supply (Hu et al., 2024a). Changes in dietary ME levels can regulate liver lipid metabolism through the coordinated modulation of lipogenesis and fatty acid oxidation pathways, thereby affecting energy storage and utilization (Hu et al., 2024b). Excessive or imbalanced energy intake can promote the generation of reactive oxygen species and activate inflammatory signaling pathways, whereas an appropriate energy supply contributes to the maintenance of redox homeostasis and immune stability (Lauridsen, 2019). The gastrointestinal tract plays a crucial role in mediating the effects of dietary energy on host metabolism. Dietary energy levels can influence the expression and activity of intestinal nutrient transporters, thereby altering the efficiency of nutrient absorption and utilization. Furthermore, increasing evidence indicates that dietary energy is a key factor regulating gut microbial composition and metabolic activity (Ban et al., 2025; Guo et al., 2025). Changes in energy levels can reshape the microbial community structure and alter microbial metabolites, which in turn regulate host lipid metabolism, immune responses, and intestinal function. This host–microbiota interaction provides an additional regulatory mechanism through which dietary energy influences physiological outcomes (Wang et al., 2026).

At present, most studies on dietary energy in broilers have primarily focused on growth performance, while integrative analyses incorporating metabolic, intestinal, and microbial responses remain limited. Given that broilers can maintain similar growth performance under different energy levels by adjusting feed intake and feed conversion efficiency (Toghyani et al., 2024), the underlying adaptive mechanisms remain poorly understood. We hypothesized that an appropriate dietary ME level would maintain metabolic homeostasis, antioxidant capacity, intestinal function, and gut microbial balance, whereas excessive dietary ME would impose metabolic stress and disrupt host physiological homeostasis. Therefore, this study established four graded dietary ME levels (3050, 3150, 3250, and 3350 kcal/kg) for broilers from 21 to 42 days of age, and systematically evaluated their effects on growth performance, metabolic status, antioxidant capacity, inflammatory responses, intestinal function, and gut microbiota composition to provide a comprehensive understanding of the physiological responses to different dietary energy levels.

## Materials and methods

### Birds, experimental design, and diets

All experiments were carried out following the guidelines set by the Institutional Animal Care and Use Committees of Henan Agricultural University (Approval No. HENAU-2022-015), and were conducted in accordance with the Guide for the Care and Use of Laboratory Animals.

A total of 480 one-day-old male AA broiler chicks were used in a 42-d feeding trial. From 1 to 21 d of age, all birds were fed a common starter diet. At 21 d of age, birds were individually weighed and randomly allocated according to body weight into four dietary treatments, with 8 replicate cages per treatment and 15 birds per replicate. Experimental diets containing four graded levels of ME (3050, 3150, 3250, and 3350 kcal/kg) were provided from 21 to 42 d of age. The ME level of 3150 kcal/kg, commonly used as the industry standard in broiler production, was set as the reference control level. The first 5 d after the dietary transition (21 to 26 d of age) were considered an adaptation period to allow birds to adjust to the experimental diets and housing conditions. At the end of the adaptation period, birds were weighed again at 26 d of age, and this body weight was used as the initial BW for the formal experimental period. Growth performance was evaluated from 26 to 42 d of age, including ADG, ADFI, and feed conversion ratio (FCR). At 42 d of age, biological samples were collected for further analyses. Diets were formulated according to commercial feeding standards, and nutrient composition met or exceeded breeder recommendations. Dietary ME levels were adjusted by modifying duck oil inclusion while maintaining similar crude protein, digestible lysine, and essential amino acid ratios among treatments.

Ingredient composition and calculated nutrient levels are presented in Table 1. Birds were housed in a commercial cage facility under standard management conditions with ad libitum access to feed and water throughout the experiment.

**Table 1:** Feed formulas for each group.

Ingredients	1-20d	21-42d			
		3050 (kg/1000kg)	3150 (kg/1000kg)	3250 (kg/1000kg)	3350 (kg/1000kg)
Corn	515	259.2	234.2	209.3	185.3
Wheat	80	350	350	350	350
Wheat Flour	—	100	100	100	100
Duck Oil	—	22	42	63	83
Soybean Oil	22	—	—	—	—
Soybean Meal (46% CP)	300	155	160	164	168
Peanut Meal (50% CP)	—	30	30	30	30
Corn Gluten Meal (60% CP)	35	20	20	20	20
Whole Duck Meal (48% CP)	—	22	22	22	22
Salt	3	2.5	2.5	2.5	2.5
Limestone Powder	14.5	10.5	10.5	10.4	10.4
Dicalcium Phosphate	18	7.8	7.9	8	8
Choline Chloride (60%)	—	0.8	0.8	0.8	0.8
Lysine (70%)	2.5	10.3	10.2	10.1	10
Methionine (99%)	3.2	2.1	2.1	2.1	2.2
Threonine	1.2	3	3	3	3
Sodium Bicarbonate	1.6	1	1	1	1
M2102 Mineral Premix 1	2	2	2	2	2
V2132 Vitamin Premix 2	2	1.8	1.8	1.8	1.8
Calculated nutrient composition					
Crude protein (%)	20.00	20.00	20.00	20.00	20.00
Crude fat (%)	4.5	4.76	6.65	8.54	10.42
Calcium (%)	0.9	0.75	0.75	0.75	0.75
Total phosphorus (%)	0.67	0.60	0.59	0.59	0.59
Available phosphorus (%)	0.45	0.38	0.38	0.38	0.38
Digestible lysine (%)	1.28	1.25	1.25	1.25	1.25
Digestible methionine (%)	0.50	0.46	0.46	0.46	0.46
Digestible methionine + cystine (%)	0.77	0.71	0.71	0.71	0.71
Digestible threonine (%)	0.86	0.84	0.84	0.84	0.84

Note:

All diets were isonitrogenous (20.00% crude protein) with balanced digestible amino acid profiles. All listed calculated nutrient values were obtained from commercial feed formulation software.

1 M2102 mineral premix was included in all diets at 2 kg/ton, supplying the following trace minerals per kilogram of complete feed: iron, 80 mg; copper, 8.0 mg; manganese, 110 mg; zinc, 65 mg; iodine, 1.1 mg; and selenium, 0.3 mg, thereby ensuring an adequate provision of trace minerals.

2 V2132 vitamin premix was supplemented at 2 kg/ton during the 1–21 d phase and at 1.8 kg/ton during the 22–42 d phase. At an inclusion rate of 2 kg/ton, the premix provided the following vitamins per kilogram of diet: vitamin A, 12,000 IU; vitamin D₃, 2,500 IU; vitamin E, 20 IU; menadione (vitamin K₃), 1.3 mg; thiamine, 2.21 mg; riboflavin, 7.8 mg; nicotinamide, 40 mg; calcium pantothenate, 16.5 mg; pyridoxine, 4 mg; biotin, 0.04 mg; folic acid, 1.2 mg; and vitamin B₁₂, 0.015 mg.

### Growth performance

Body weight and feed intake were recorded on a replicate basis at 26 and 42 d of age. ADG, ADFI, and FCR were calculated for the period from 26 to 42 d. Mortality was recorded daily, and FCR was calculated and corrected for mortality.

### Sample collection

At 42 d of age, one bird per replicate was randomly selected after 12 h feed withdrawal. Blood samples were collected from the wing vein, centrifuged at 3,000 × g for 10 min at 4°C, and the serum was stored at -80°C until analysis. The pancreas, proventriculus, gizzard, liver, thymus, spleen, and bursa of Fabricius were carefully excised, rinsed with ice-cold saline, gently blotted dry with filter paper, and weighed individually. Organ index was calculated as organ weight (g) divided by live body weight (g) × 100. Liver and ileal tissue were collected, snap-frozen in liquid nitrogen, and stored at −80°C for subsequent analyses. Cecal digesta samples were aseptically collected, immediately frozen in liquid nitrogen, and stored at -80°C for microbiota analysis.

### Serum inflammatory cytokine and oxidative stress analysis

Serum concentrations of inflammatory cytokines, interleukin-1β (IL-1β), interleukin-6 (IL-6), interleukin-10 (IL-10), interferon-γ (IFN-γ) and tumor necrosis factor-α (TNF-α) were measured using commercial ELISA kits (Meimian Biotechnology Co., Ltd., Yancheng, China) according to the manufacturer's instructions. Oxidative stress-related parameters, including superoxide dismutase (SOD), catalase (CAT), glutathione peroxidase (GSH-Px), myeloperoxidase (MPO) and hydrogen peroxide(H₂O₂) were determined using colorimetric assay kits (Nanjing Jiancheng Bioengineering Institute, Nanjing, China) following standard protocols.

Spearman correlation analysis was performed to evaluate the associations between immune organ indices and serum immune-related parameters, including inflammatory cytokines and antioxidant indicators. Correlation coefficients were calculated, and statistical significance was determined at *P* < 0.05.

### RNA extraction and quantitative real-time PCR

Total RNA was extracted from liver and intestinal tissues using TRIzol reagent (Nanjing Vazyme Biotech Co., Ltd., Nanjing, China). RNA concentration and purity were measured using a micro-spectrophotometer (Thermo Fisher Scientific, Wilmington, DE, USA), and RNA integrity was verified by agarose gel electrophoresis. First-strand cDNA was synthesized using a reverse transcription kit (Nanjing Vazyme Biotech Co., Ltd., Nanjing, China). Quantitative real-time PCR (RT-qPCR) was performed using SYBR Green Master Mix (Nanjing Vazyme Biotech Co., Ltd., Nanjing, China) on a real-time PCR detection system (Thermo Fisher Scientific, USA). The PCR reaction volume was 20 μL, containing cDNA template, SYBR Green Master Mix, and gene-specific primers. The thermal cycling conditions were as follows: initial denaturation at 95°C for 30 s, followed by 40 cycles of 95°C for 10 s and 60°C for 30 s. Melting curve analysis was conducted to confirm amplification specificity. Relative gene expression levels were calculated using the 2^−ΔΔCt method with β-actin as the internal reference gene. Primer sequences are listed in Table S1.

### Cecal microbiota analysis

Microbial genomic DNA was extracted from cecal digesta using a commercial DNA extraction kit (Tiangen Biotech Co., Ltd., Beijing, China) according to the manufacturer’s instructions. DNA quality and concentration were assessed using a NanoDrop spectrophotometer (Thermo Fisher Scientific, Waltham, MA, USA). The V3–V4 regions of the bacterial 16S rRNA gene were amplified using universal primers (F: ACTCCTACGGGAGGCAGCA; R: GGACTACHVGGGTWTCTAAT). The obtained PCR products were purified using the AxyPrep DNA Gel Extraction Kit (Axygen Biosciences, Union City, CA, United States), and sequencing was performed on an Illumina MiSeq platform (Personal Biotechnology Co., Ltd., Shanghai, China).

Raw reads were processed using QIIME 2 (version 2019.4). After quality filtering, paired-end reads were merged and chimeric sequences were removed. Operational taxonomic units (OTUs) were clustered at 97% sequence similarity. Representative sequences were selected for each OTU, and taxonomic assignment was performed against the Greengenes database (gg_13_8 release). Alpha diversity indices (Chao1 and Shannon) and beta diversity based on Bray–Curtis distances were calculated in QIIME 2. Principal coordinate analysis (PCoA) was used to visualize differences in microbial community structure among treatment groups. Differential taxa were identified using linear discriminant analysis effect size (LEfSe).

### Statistical analysis

All data were analyzed using IBM SPSS Statistics 26 software. Data were first tested for normality using the Shapiro–Wilk test and for homogeneity of variances using Levene’s test. When these assumptions were satisfied, data were analyzed by one-way ANOVA and differences among treatment means were compared using the SNK multiple comparison test. In addition, orthogonal polynomial contrasts were performed to evaluate the linear and quadratic responses to increasing dietary ME levels. Data were presented as mean ± SD, and statistical significance was set at *P* < 0.05.

## Results

### Growth performance

The growth performance of broilers fed diets with graded ME levels from 26 to 42 d of age is presented in Table 2. Dietary ME level had no significant effect on body weight, average daily gain, average daily feed intake and feed conversion ratio among the four treatments at 26 and 42 d of age (*P_ANOVA_* > 0.05). Meanwhile, no significant linear or quadratic responses of these growth performance indices to graded dietary ME levels were detected (all *P_linear_* > 0.05, all *P_quadratic_* > 0.05).

**Table 2:** Growth performance of broilers fed diets with graded ME levels from 26 to 42 d of age.

	Group				*P*-value	Linear	Quadratic
	3050	3150	3250	3350			
BW at 26 days (g)	901.03±60.24	874.04±65.96	914.56±40.18	926.46±32.58	0.23	0.163	0.296
BW at 42 days (g)	2528.21±157.34	2372.15±132.73	2429.13±248.04	2493.24±185.86	0.36	0.872	0.105
ADG (g)	101.70±7.48	93.63±7.65	94.66±13.15	97.92±10.43	0.38	0.518	0.119
ADFI (g)	173.01±8.42	165.79±15.62	163.79±17.77	162.09±8.45	0.39	0.108	0.561
FCR	1.71±0.09	1.78±0.16	1.74±0.15	1.67±0.13	0.42	0.494	0.134
Mortality (%)	2.50	1.67	3.33	2.50			

Data are expressed as mean ± SD (n =6); 3050 kcal/kg, 3150 kcal/kg, 3250 kcal/kg, and 3350 kcal/kg, ME: metabolizable energy; BW: Body weight; ADG: Average daily gain; ADFI: Average daily feed intake; FCR: Feed conversion ratio. Means within a row without a common superscript differ, *P* < 0.05.

### Organ indices

The effects of dietary ME level on organ indices of broilers at 42 d of age are summarized in Table 3.

**Table 3:** Organ indices of broilers fed diets with graded ME levels at 42 d of age.

Organ	Group				*P*-value	Linear	Quadratic
	3050	3150	3250	3350			
Pancreas	0.14±0.01	0.14±0.01	0.12±0.02	0.14±0.02	0.154	0.954	0.328
Proventriculus	0.58±0.05	0.59±0.05	0.56±0.19	0.54±0.02	0.872	0.521	0.680
Gizzard	1.08±0.23^b^	1.36±0.12^a^	1.04±0.26^b^	0.89±0.09^b^	0.003	0.019	0.013
Liver	2.00±0.11	1.98±0.04	2.10±0.22	1.92±0.07	0.146	0.572	0.149
Thymus	0.19±0.05^b^	0.27±0.06^a^	0.28±0.02^a^	0.34±0.09^a^	0.003	0.001	0.510
Spleen	0.10±0.01^c^	0.20±0.03^b^	0.25±0.03^a^	0.18±0.05^b^	0.001	0.001	0.001
Bursa of Fabricius	0.03±0.01^b^	0.04±0.01^a^	0.02±0.001^b^	0.03±0.001^ab^	0.008	0.680	0.602

Data are expressed as mean ± SD (n =6); 3050 kcal/kg, 3150 kcal/kg, 3250 kcal/kg, and 3350 kcal/kg.

Means within a row without a common superscript differ, *P* < 0.05.

Dietary ME level had no significant effect on the indices of the pancreas, proventriculus and liver, with no significant linear or quadratic responses observed (all *P_ANOVA_* > 0.05, *P_linear_* > 0.05, *P_quadratic_* > 0.05). Dietary ME level significantly affected the indices of the gizzard, thymus, spleen, and bursa of Fabricius. The gizzard index in the 3150 kcal/kg group was significantly higher than that in the other groups (*P_ANOVA_* = 0.003), showing both linear (*P_linear_* = 0.019) and quadratic (*P_quadratic_* = 0.013) responses to increasing dietary ME levels. Dietary ME level also significantly affected the thymus (*P_ANOVA_* = 0.003), spleen (*P_ANOVA_* = 0.001), and bursa of Fabricius (*P_ANOVA_* = 0.008) indices. The thymus index exhibited a significant linearly increasing trend with elevated dietary ME levels (*P_linear_* = 0.001), whereas the spleen index showed both linear and quadratic responses (*P_linear_* = 0.001, *P_quadratic_* = 0.001). Although the bursa of Fabricius index differed among treatments, no significant linear or quadratic trends were observed (*P_linear_* and *P_quadratic_* > 0.05).

### Serum inflammatory cytokines and antioxidant indices

**Fig. 1:**
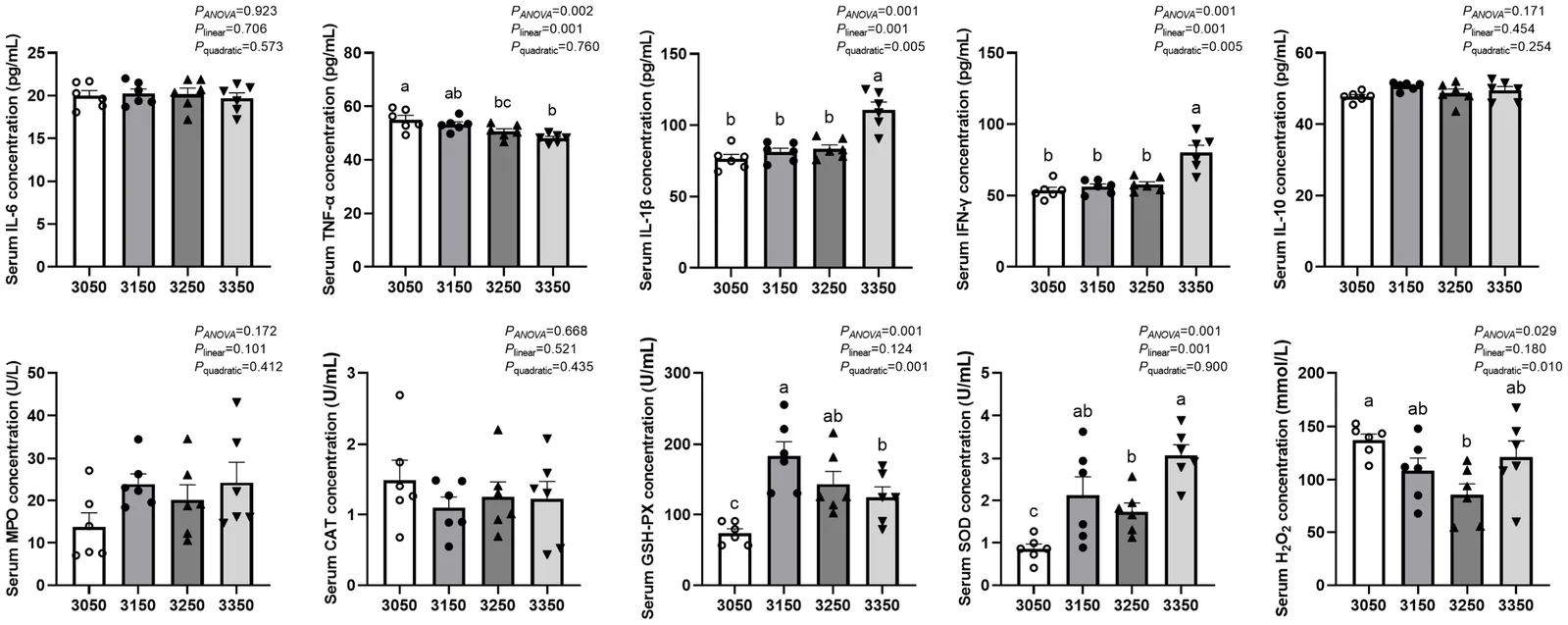
Serum inflammatory cytokines and antioxidant indices of broilers fed diets with graded ME levels at 42 d of age. Note: Means within a row without a common superscript differ, *P_ANOVA_ < 0.05*.

The effects of ME levels on serum inflammatory cytokines and antioxidant indices in broilers at 42 d of age are shown in Fig. 1.

Dietary ME levels significantly affected serum TNF-α, IL-1β, IFN-γ, GSH-Px, SOD, and H₂O₂ concentrations (*P_ANOVA_* < 0.05), whereas serum IL-6, IL-10, MPO, and CAT were not affected (*P_ANOVA_* > 0.05). Serum TNF-α decreased linearly with increasing dietary ME level (*P_linear_* = 0.001). In contrast, serum IL-1β and IFN-γ exhibited significant linear and quadratic responses (*P_linear_* < 0.001, *P_quadratic_* = 0.005), with the highest values observed in the 3350 kcal/kg group. Serum GSH-Px activity showed a significant quadratic response (*P_quadratic_* = 0.001), reaching the highest level in the 3150 kcal/kg group. Serum SOD activity increased linearly with increasing dietary ME level (*P_linear_* = 0.001). Serum H₂O₂ concentration exhibited a significant quadratic response (*P_quadratic_* = 0.010), with the lowest concentration observed in the 3250 kcal/kg group.

### Correlation analysis between immune organs and serum immune-related parameters

Spearman correlation analysis was performed to assess the associations between immune organ indices and serum inflammatory and antioxidant parameters (Fig. S1). Significant correlations were observed between immune organ indices and serum indicators. The thymus index was positively correlated with IL-1β, IFN-γ, and TNF-α, but negatively correlated with GSH-Px and CAT. The liver index showed negative correlations with IL-1β and IFN-γ, whereas the spleen index was negatively correlated with H_2_O_2_ levels.

### Liver expression of genes related to glucose and lipid metabolism

**Fig. 2:**
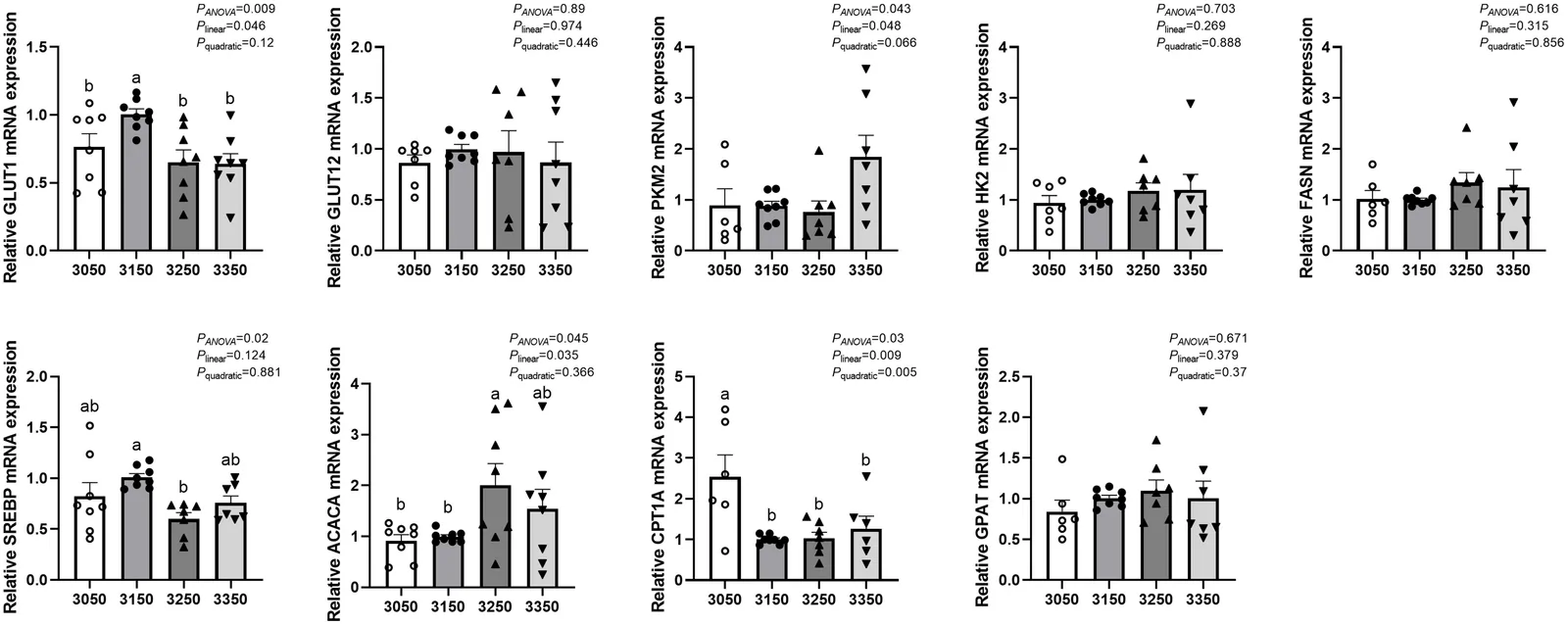
Liver expression of genes related to glucose and lipid metabolism in broilers fed diets with graded ME levels at 42 d of age. Note: Means within a row without a common superscript differ, *P_ANOVA_ < 0.05*.

**Fig. 3:**
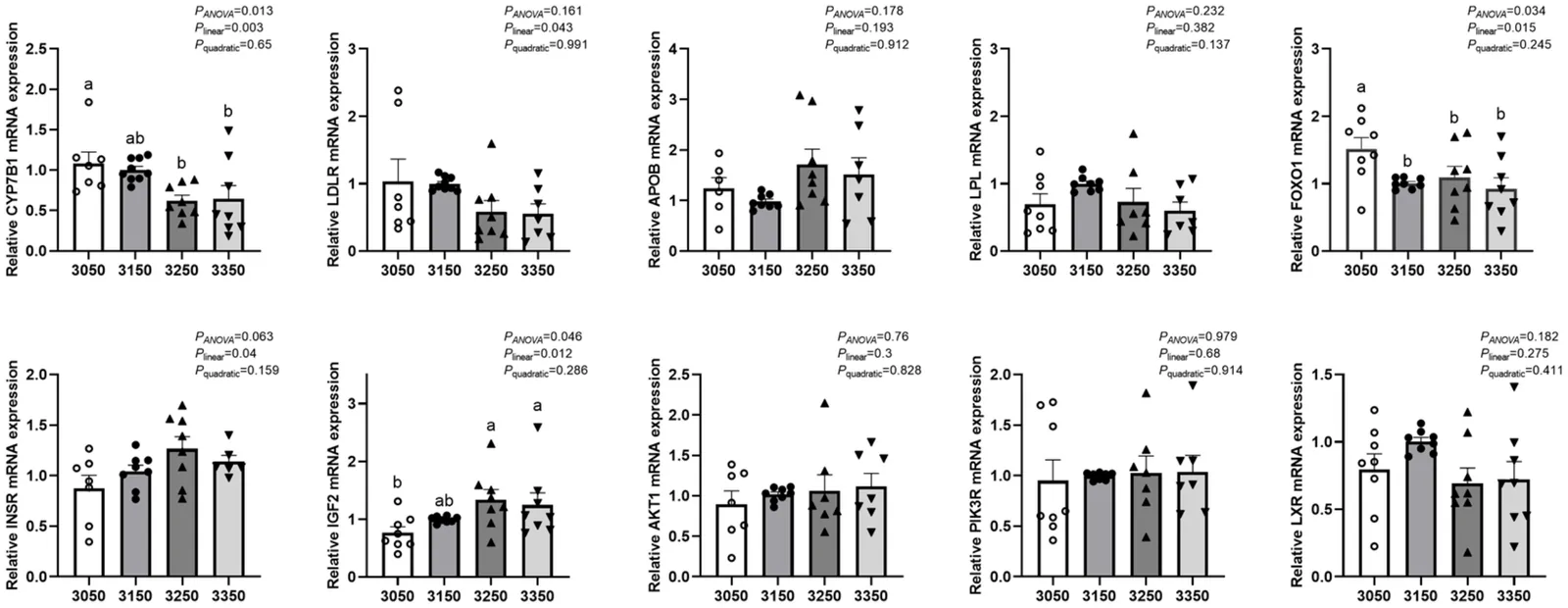
Liver expression of genes related to lipid transport, cholesterol metabolism, and insulin signaling in broilers fed diets with graded ME levels at 42 d of age. Note: Means within a row without a common superscript differ, *P_ANOVA_ < 0.05*.

The effects of dietary ME levels on liver gene related to glucose and lipid metabolism in broilers at 42 d of age are shown in Fig. 2, Fig. 3.

For glucose metabolism, the expression of glucose transporter 1 (GLUT1) was significantly affected by dietary ME levels (*P_ANOVA_* = 0.009) and exhibited a significant linear response to increasing dietary ME levels (*P_linear_* = 0.046). With increasing dietary ME levels, the expression of pyruvate kinase M2 (PKM2) was significantly upregulated in the 3350 kcal/kg group (*P_ANOVA_* = 0.043, *P_linear_* = 0.048). In contrast, the expression levels of glucose transporter 12 (GLUT12) and hexokinase 2 (HK2) did not differ among treatments (*P_ANOVA_* > 0.05).

For lipid metabolism and transport, the expression of genes involved in lipid synthesis and transport was regulated by dietary ME. When ME increased to 3250 kcal/kg, the expression of sterol regulatory element-binding protein (SREBP) was significantly lower than that in the 3150 kcal/kg group (*P_ANOVA_* < 0.05), whereas acetyl-CoA carboxylase alpha (ACACA) expression was significantly higher than that in the 3050 and 3150 kcal/kg groups (*P_ANOVA_* < 0.05), and increased linearly with increasing dietary ME (*P_linear_* = 0.035). The expression of carnitine palmitoyl transferase 1A (CPT1A) was significantly influenced by dietary ME levels (*P_ANOVA_* = 0.030) and exhibited both significant linear (*P_linear_* = 0.009) and quadratic (*P_quadratic_* = 0.005) responses. With increasing dietary ME levels, the expression of cytochrome P450 family 7 subfamily B1 (CYP7B1) and low-density lipoprotein receptor (LDLR) showed a decreasing trend (*P_linear_* < 0.05). The expression levels of fatty acid synthase (FASN), glycerol-3-phosphate acyltransferase (GPAT), apolipoprotein B (APOB), and lipoprotein lipase (LPL) were not affected by dietary ME levels (*P_ANOVA_* > 0.05).

### Liver expression of genes related to insulin signaling pathway

The effects of dietary ME levels on liver gene related to insulin signaling pathway in broilers at 42 d of age are shown in Fig. 3.

Regarding insulin signaling and growth-related genes, the expression of forkhead box protein O1 (FOXO1) was highest in the 3050 kcal/kg group (*P_ANOVA_* = 0.034) and exhibited a significant linear response (*P_linear_* = 0.015). The expression levels of insulin receptor (INSR) and insulin-like growth factor 2 (IGF2) tended to increase with increasing dietary ME (*P_linear_* < 0.05). In contrast, the expression of AKT serine/threonine kinase 1 (AKT1), phosphoinositide-3-kinase regulatory subunit (PIK3R), and liver X receptor (LXR) was not significantly affected by dietary ME (*P_ANOVA_* > 0.05).

### Ileal gene expression

**Fig. 4:**
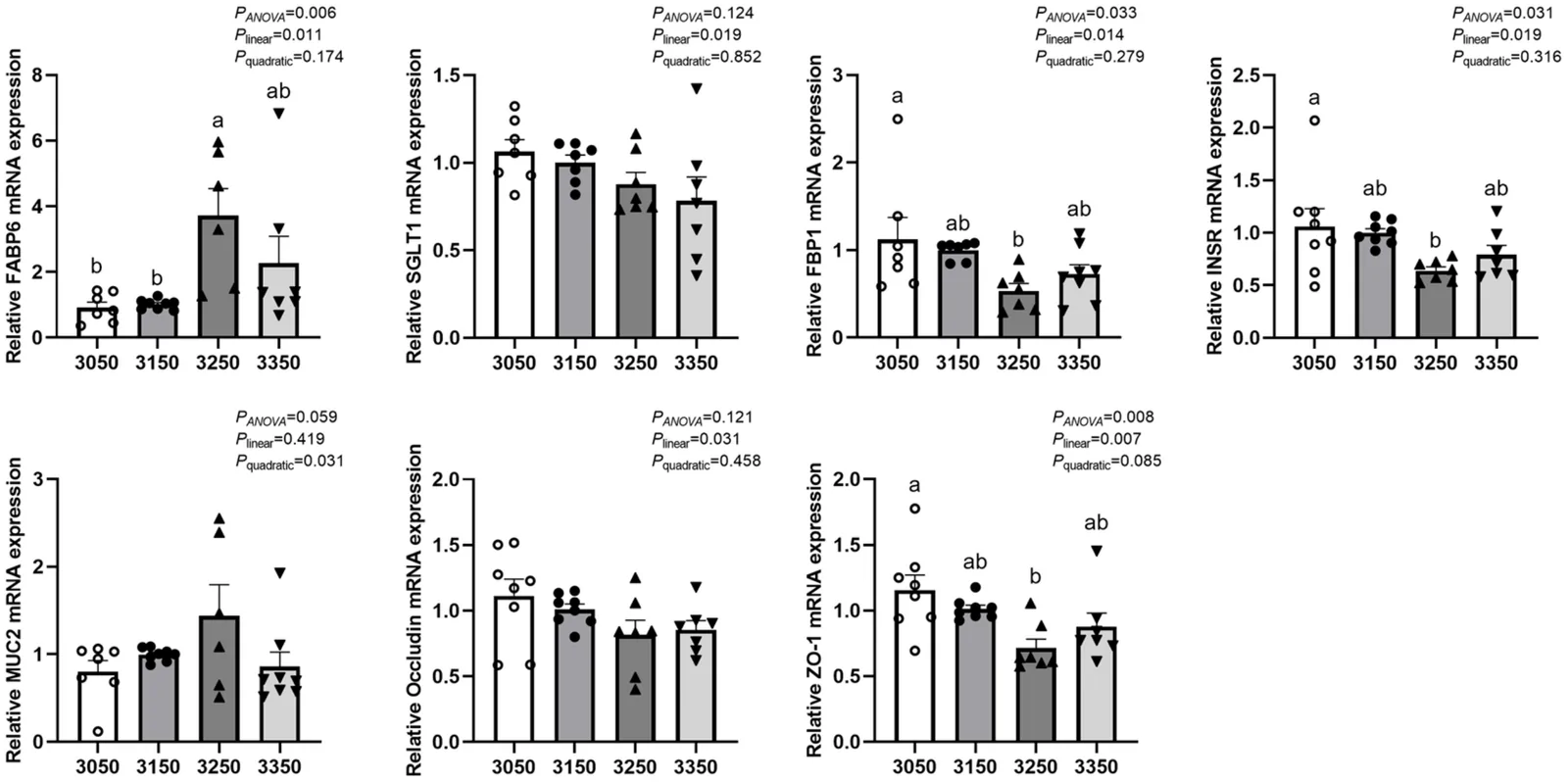
Intestinal gene expression of broilers fed diets with graded ME levels at 42 d of age. Note: Means within a row without a common superscript differ, *P_ANOVA_ < 0.05*.

The effects of dietary ME levels on ileal gene expression in broilers at 42 d of age are presented in Fig. 4.

Dietary ME influenced the expression of genes related to nutrient transport and metabolism. The expression of fatty acid binding protein 6 (FABP6), fructose-1,6-bisphosphatase 1 (FBP1), insulin receptor (INSR), and ZO-1 was significantly affected by dietary ME levels (*P_ANOVA_* < 0.05). As dietary ME increased, FABP6 expression increased linearly (*P_linear_* < 0.05), whereas FBP1, INSR, and ZO-1 expression decreased linearly (*P_linear_* < 0.05). Compared with the 3050 and 3150 kcal/kg groups, FABP6 expression was significantly higher in the 3250 kcal/kg group (*P_ANOVA_* < 0.05), while FBP1, INSR, and ZO-1 expression was significantly lower (*P_ANOVA_* < 0.05). The expression of MUC2 and Occludin was not significantly affected by dietary ME levels (*P_ANOVA_* > 0.05), while a significant quadratic response was detected for MUC2 (*P_quadratic_* = 0.031).

### Cecal microbiota

**Fig. 5:**
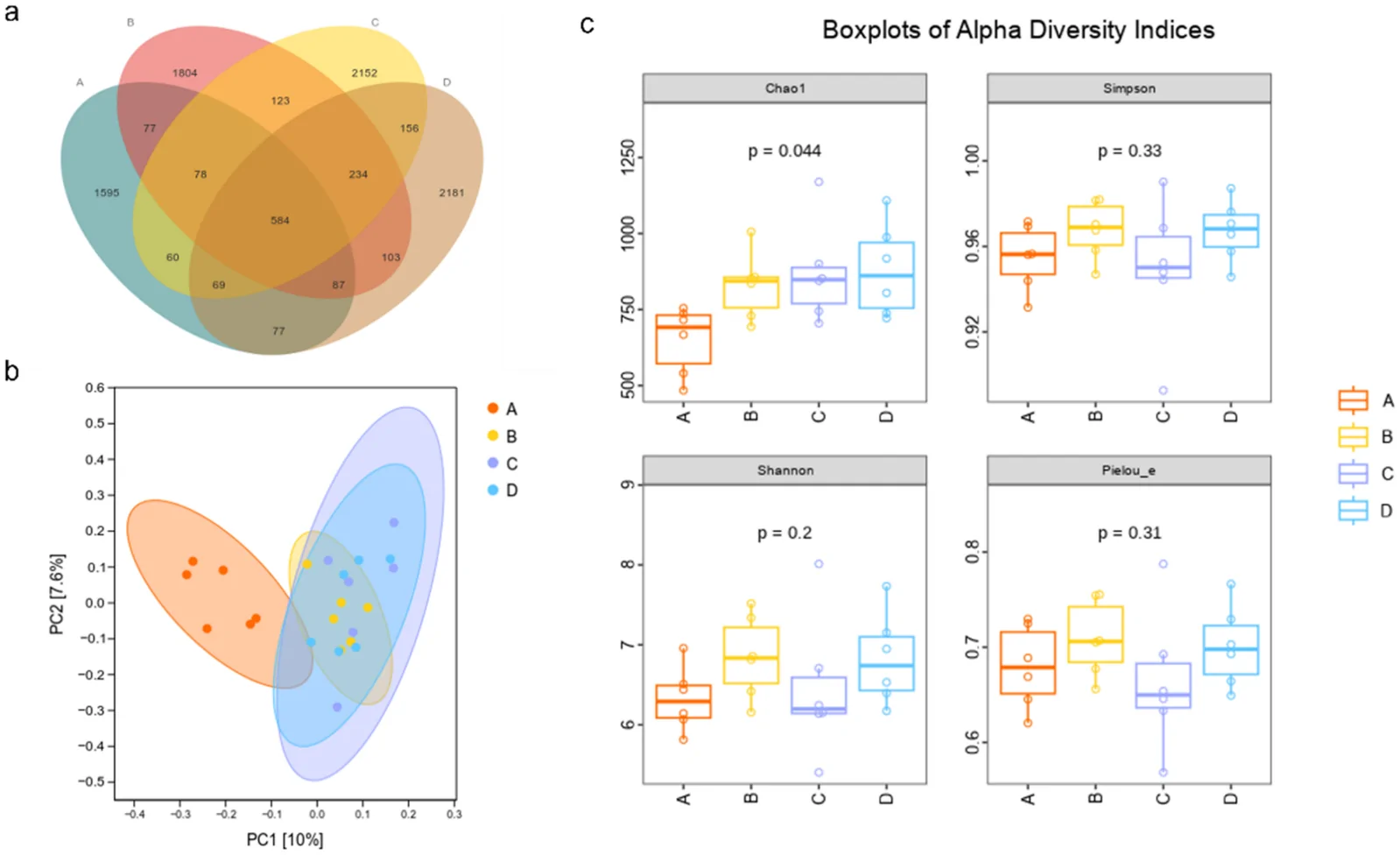
Effects of graded dietary ME levels on α-diversity and community structure of cecal microbiota in broilers. (a) Venn diagram of cecal microbiota OTUs. (b) Principal co-ordinates analysis (PCoA) plot of OTU levels of bacterial communities based on bray_curtis distance. (c) Shannon and Simpson indices representing microbial community diversity; Chao 1 index representing microbial community richness. Pielou_e index representing community evenness. Groups: A (3050 kcal/kg), B (3150 kcal/kg), C (3250 kcal/kg), and D (3350 kcal/kg).

A total of 10,401 OTUs were identified across all samples. The number of unique OTUs varied among treatments, with 1,595, 1,804, 2,152, and 2,181 OTUs detected in the 3050, 3150, 3250, and 3350 kcal/kg groups, respectively (Fig. 5a). PCoA based on Bray–Curtis distances showed limited separation of cecal microbial communities among the four treatments (Fig. 5b). Alpha diversity analysis showed that the Chao1 index differed significantly among treatments (*P_ANOVA_* = 0.044), whereas no significant differences were observed in Shannon and Simpson indices, and Pielou’s evenness (Fig. 5c).

**Fig. 6:**
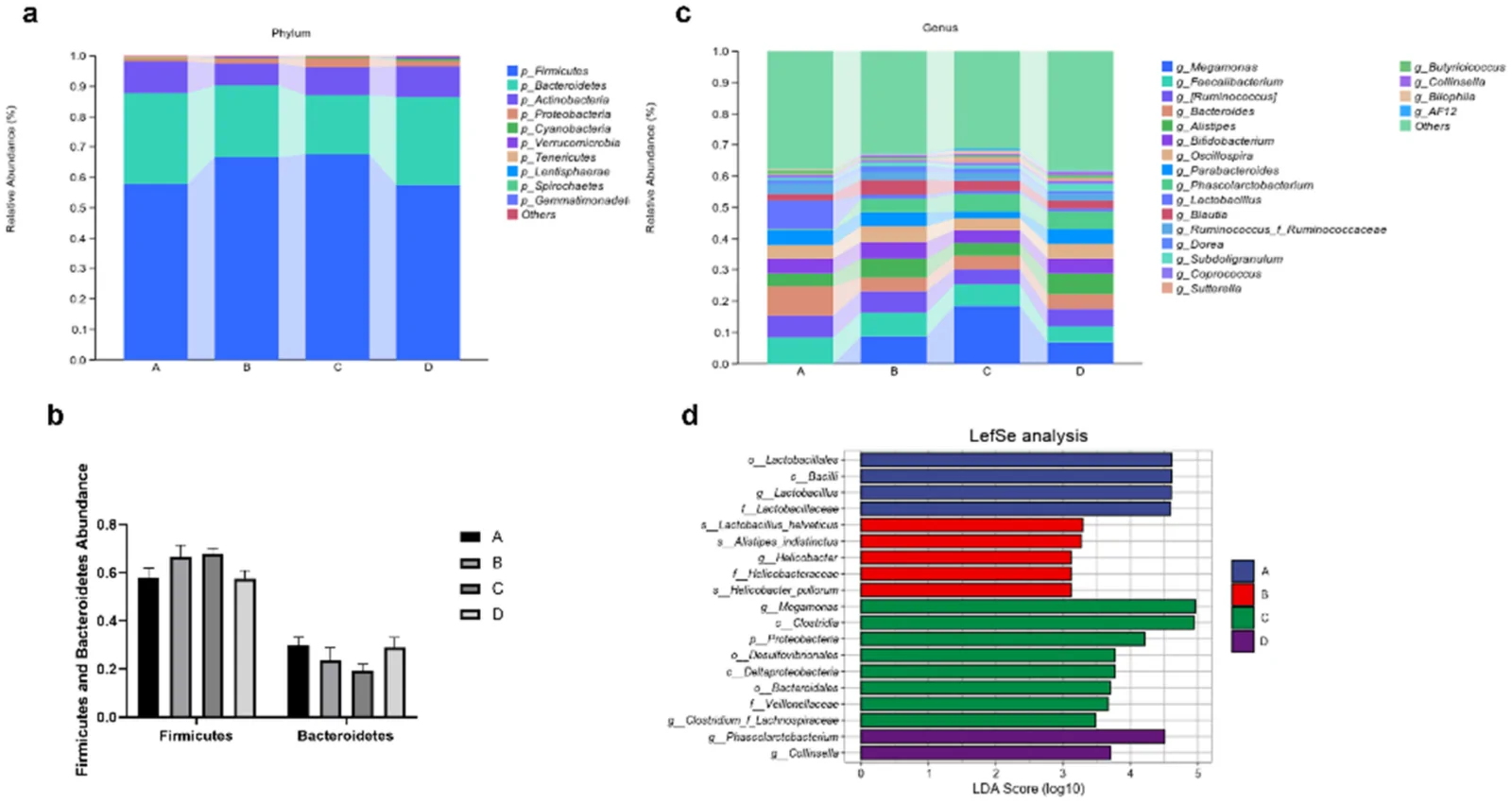
Effects of graded dietary ME levels on the composition of cecal microbiota at the phylum and genus levels in broilers. (a) Relative abundance of dominant bacterial phyla in each group. (b) Relative abundance of Firmicutes and Bacteroidetes.(c) Relative abundance of dominant bacterial genera in each group. (d) Identification of significantly different taxa among groups using linear discriminant analysis effect size (LEfSe) with default parameters. Groups: A (3050 kcal/kg), B (3150 kcal/kg), C (3250 kcal/kg), and D (3350 kcal/kg).

At the phylum level, Firmicutes and Bacteroidetes were the dominant bacterial phyla across all groups (Fig. 6a). The relative abundance of *Firmicutes* was higher in the 3150 and 3250 kcal/kg groups compared with the 3050 and 3350 kcal/kg groups, whereas *Bacteroidetes* showed the opposite pattern (Fig. 6b). At the genus level, *Megamonas, Faecalibacterium, Ruminococcus, Bacteroides, Alistipes, Blautia, Lactobacillus*, and *Ruminococcaceae* were the predominant genera (Fig. 6c). The 3250 kcal/kg group exhibited a higher relative abundance of *Megamonas* compared with the other treatments.

LEfSe analysis revealed distinct microbial biomarkers among treatments (Fig. 6d). The 3050 kcal/kg group was enriched in *Lactobacillales*-related taxa, including *Bacilli, Lactobacillaceae*, and *Lactobacillus*. The 3150 kcal/kg group was characterized by enrichment of *Helicobacter*. The 3250 kcal/kg group showed higher abundance of *Megamonas, Clostridia, Proteobacteria, Desulfovibrionales, Bacteroidales*, and *Phascolarctobacterium*. In contrast, *Collinsella* was the primary enriched genus in the 3350 kcal/kg group.

## Discussion

The present study evaluated the effects of graded dietary ME from 21 to 42 d of age on growth performance, organ development, systemic oxidative and inflammatory status, liver and intestinal gene expression, and cecal microbiota in broilers. Overall, increasing dietary ME within this range did not significantly influence body weight, ADG, ADFI, or FCR, but it modulated immune organ indices, antioxidant capacity, metabolic gene expression, and microbial community composition.

Traditional studies have suggested that high-energy diets can improve feed conversion efficiency by reducing feed intake. However, recent research on ME and poultry growth performance indicates that the effects of dietary energy levels on growth outcomes are inconsistent. Previous studies have shown that birds fed diets with energy levels above the standard exhibited reduced ADFI (Abouelezz et al., 2019). Similarly, increasing dietary energy levels was reported to improve final body weight and FCR, whereas other studies found that low-energy diets could increase body weight (Houshmand et al., 2011; Yu et al., 2024). Relevant studies have shown that diets containing approximately 3000 kcal/kg ME had no significant effect on body weight, while low-energy diets reduced body weight and high-energy diets decreased ADFI (Ferreira et al., 2015). These inconsistent findings may be related to differences in genotype, age, dietary composition, and feeding conditions. Although dietary ME levels differed, crude protein, digestible lysine, and essential amino acid ratios were maintained at similar levels, reducing nutritional limitations other than energy. In addition, broilers can partially compensate for differences in dietary energy density by regulating voluntary feed intake, thereby maintaining comparable growth performance. Furthermore, the dietary ME range evaluated (3050–3350 kcal/kg) may have remained within the physiological adaptive capacity of broilers, resulting in limited effects on growth traits.

Immune organs play critical roles in both cellular and humoral immunity, and their relative weights are commonly used indicators for evaluating immune status. Previous studies have demonstrated that dietary energy levels and nutritional interventions can influence the development of immune organs and immune responses in broilers (Hu et al., 2022; Omara et al., 2021; Shi et al., 2022). In this study, dietary ME levels had no significant effect on growth performance parameters, whereas significant differences were observed in the indices of the thymus, spleen, and bursa of Fabricius among treatments. The thymus index showed a linear increase with increasing dietary ME levels, while the spleen index exhibited both linear and quadratic responses, suggesting that immune organ development was sensitive to dietary energy availability (García-Gómora et al., 2024). In addition to immune organs, dietary ME levels also influenced the indices of digestive organs. The gizzard index was significantly affected by dietary ME levels and showed a quadratic response, with the highest value observed in the 3150 kcal/kg group. In contrast, the indices of pancreas and proventriculus were not significantly affected. These results suggest that different dietary energy levels may induce adaptive changes in digestive organ development, while maintaining relatively stable growth performance (Perween et al., 2016).

Serum inflammatory cytokine analysis showed a non-uniform pattern across dietary ME levels, rather than a consistent dose-dependent trend. Elevated levels of multiple inflammatory cytokines were observed in both the 3050 and 3350 kcal/kg groups, suggesting that either insufficient or excessive energy supply may alter the systemic inflammatory status (Jiang et al., 2025; Wang, Zhou, Abouelezz, Cao, Shi and Hou, 2026). In contrast, antioxidant indices exhibited a clearer response pattern. Increasing dietary ME levels generally improved antioxidant status, although responses varied among individual antioxidant parameters, which is consistent with previous findings (Mao et al., 2024; Tang et al., 2019). However, no further improvement in oxidative stress markers was observed in the 3350 kcal/kg group, indicating that excessive energy intake does not confer additional antioxidant benefits and may even increase the metabolic burden.

Furthermore, Spearman correlation analysis showed significant associations between immune organ indices and serum immune-related parameters. The thymus index was positively correlated with IL-1β, IFN-γ, and TNF-α, but negatively correlated with GSH-Px and CAT, suggesting that thymus development was associated with changes in inflammatory and antioxidant status under different dietary ME levels (Kidd, 2004). In addition, the liver index was negatively correlated with IL-1β and IFN-γ, while the spleen index was negatively correlated with H₂O₂ levels. These results indicate that dietary ME levels may be related to immune organ development and systemic immune responses, although further studies are needed to clarify the underlying mechanisms (Surai et al., 2019).

The liver, as the primary organ for lipid synthesis, exhibits a pronounced transcriptional response to dietary ME levels. Changes in dietary ME levels altered the expression of genes associated with hepatic glucose metabolism. GLUT1 expression was significantly affected by dietary ME levels and exhibited a significant linear response to increasing dietary ME levels, whereas PKM2 expression was significantly upregulated in the 3350 kcal/kg group, suggesting that glucose metabolism-related transcriptional regulation may be involved in hepatic responses to variations in energy supply (Liu et al., 2020). Regarding lipid metabolism, dietary ME levels affected the expression of genes associated with hepatic lipid synthesis, fatty acid oxidation, and transport. The altered expression of lipogenesis-related genes under different ME conditions suggested that dietary energy availability may regulate hepatic lipid metabolic pathways at the transcriptional level (Chen et al., 2023a; Chen et al., 2023b; Geng and Guo, 2024). Under lower dietary ME levels, the increased expression of fatty acid catabolism-related genes indicated a potential enhancement of lipid metabolism-related regulatory responses to maintain energy homeostasis (Cogburn et al., 2020; Hu et al., 2024c). Meanwhile, genes involved in cholesterol metabolism and lipoprotein transport also showed corresponding changes, suggesting that dietary energy levels may modulate hepatic lipid metabolic regulation through transcriptional mechanisms (Guo et al., 2023; Li et al., 2024).

Insulin signaling and growth-related genes also exhibited adaptive regulatory patterns. Increasing dietary ME significantly upregulated IGF2 expression while downregulating FOXO1 expression, suggesting transcriptional modulation of insulin signaling-related pathways under different energy supplies. In contrast, AKT1 and PIK3R expression remained relatively stable, suggesting that the transcriptional responses of these downstream signaling components were comparatively stable. Collectively, these findings suggest that broilers may adapt to variations in energy supply through coordinated transcriptional regulation of glucose metabolism, lipid metabolism and transport, and insulin signaling pathways.

In terms of intestinal function, the expression of FABP6 increased with rising dietary ME levels and reached a peak in the 3250 kcal/kg group, whereas FBP1 and INSR exhibited opposite patterns. These results suggest that higher dietary energy density may regulate genes associated with intestinal bile acid and lipid transport, while also affecting glucose metabolism and insulin signaling-related transcriptional responses (Sadr et al., 2025; Wang et al., 2024). In contrast, among intestinal barrier–related genes, the expression level of ZO-1 decreased with increasing dietary ME levels, while MUC2 and Occludin showed no significant changes among all groups. Indicating that energy levels within the tested range had limited effects on the transcriptional expression of intestinal barrier-related genes. Overall, intestinal responses to dietary energy variation were mainly characterized by transcriptional regulation rather than alterations in barrier-related gene expression.

Further analysis of the intestinal microbiota revealed that, except for the Chao1 index, most α-diversity indices did not change significantly, and β-diversity analysis showed low separation among treatment groups. These results indicate that the dietary ME levels applied in this study did not induce large-scale changes in overall cecal microbial community structure, but rather modulated the abundance of specific microbial taxa. At the phylum level, the relative abundances of Firmicutes and Bacteroidetes varied among groups, with a higher abundance of Firmicutes observed in the medium-ME group. Firmicutes and Bacteroidetes are dominant bacterial phyla in the avian gut and have been widely associated with nutrient metabolism and energy-related microbial functions. Therefore, the altered abundance of these bacterial groups may reflect changes in microbial community composition in response to different dietary energy supplies rather than direct changes in microbial metabolic activity. At the genus level, dietary ME levels markedly affected several specific bacterial populations. The abundance of *Megamonas* and *Collinsella* increased in the high-ME group, suggesting that higher dietary energy availability may favor the enrichment of microbial populations associated with carbohydrate metabolism (Guo, Liu, Yang, Gao, Zhang, Yang and Xin, 2025). Collinsella has also been reported to be associated with host metabolic traits, including glucose and lipid metabolism (Liu et al., 2023), indicating that its altered abundance may represent a potential microbial response to changes in dietary energy supply. In contrast, *Lactobacillus* was enriched in the 3050 kcal/kg group, which may represent a microbial compositional response to differences in dietary energy availability. Lactobacillus has been reported to be associated with carbohydrate utilization and intestinal homeostasis (Deng et al., 2021). Microbial metabolites such as short-chain fatty acids (SCFAs) are important products of intestinal microbial fermentation and may participate in host metabolic regulation. Although SCFAs were not measured in the present study, changes in microbial taxa associated with carbohydrate metabolism may provide potential links between dietary energy supply and host metabolic responses. Further studies integrating microbial metabolite analysis or metagenomic approaches are needed to clarify the functional significance of these microbial alterations. Collectively, dietary ME levels primarily influenced the relative abundance of specific cecal microbial taxa rather than causing extensive microbial community restructuring. These microbial shifts, together with alterations in host metabolic responses, suggest that gut microbiota may participate in the adaptive response of broilers to different dietary energy supplies.

## Conclusion

Taken together, the present findings indicate that graded dietary ME levels ranging from 3050 to 3350 kcal/kg had limited effects on growth performance but influenced immune organ development, antioxidant capacity, liver metabolic gene expression, and the relative abundance of specific cecal microbial taxa. Among the tested ME levels, the 3250 kcal/kg group exhibited relatively favorable antioxidant responses and coordinated regulation of liver metabolic-related gene expression, although further validation is required to determine whether these transcriptional changes translate into functional metabolic improvements. Increasing dietary energy beyond this level did not enhance growth efficiency and was associated with distinct shifts in both metabolism and microbial composition.

These results highlight that optimizing dietary ME for modern broilers should consider not only growth performance but also physiological regulation, antioxidant status, and microbial responses. Further studies integrating nutrient partitioning, metabolic profiling, and gut–liver interactions are warranted to refine dietary energy recommendations for broiler production.

## Funding

This study was supported by grants from the 2025 Henan Provincial Major Science and Technology Project (251100110400).

## Disclosures

The authors declare that they have no known competing financial interests or personal relationships that could have appeared to influence the work reported in this paper.

## Appendix Supplementary materials

Image, application 1

## Footnotes

Supplementary material associated with this article can be found, in the online version, at doi:10.1016/j.psj.2026.107520.
