# Supplementary material for: Effects of different dietary metabolizable energy levels on growth performance, liver metabolism, and gut microbiota in growing broilers
Source: Poult Sci. 2026 Jul 28;105(11):107520. doi: 10.1016/j.psj.2026.107520 (PMC13499402; doi:10.1016/j.psj.2026.107520)
Supplement: Supplementary file 1 [file mmc1.docx]

Table S1 Gene Primer Sequences

| Gene | Forward Primer | Reverse Primer | Product Length | |  |
| --- | --- | --- | --- | --- | --- |
| β-actin | GTCCACCGCAAATGCTTCTAA | TGCGCATTTATGGGTTTTGTT | | 78 | |
| CYP7B1 | TGCGTGACGAGATTGACCAT | TCGTTTAAGGCGCTCTCCAG | | 121 | |
| APOB | GCAGCTTTGCTCATCGTGAC | AACGTCAGCAAATGTTGGGC | | 119 | |
| LDLR | TTCGAGGACTCCGTGTTCTG | GCAGAGATTCGGCCACGAC | | 100 | |
| LPL | GCTCACTACCGAGACATGGG | GACCGGACGGTTTCAGTTCT | | 92 | |
| INSR | CATGGTAGGGGCAGCTGAG | CCCACTCTTGGGTATCGCTG | | 113 | |
| AKT1 | AGCCTCCTCCTCTCGGC | GGCTCCTCCTCCCCTTCTC | | 112 | |
| PIK3R | CTTCACTGACTGCCTCGGAC | AGGAGGTAACCACGGCAATG | | 74 | |
| FOXO1 | CGGTTGGACTGTGACATGGA | ACCCAACTGTGTGTGGTTGT | | 134 | |
| IGF2 | TCAGTAGACCAGTGGGACGA | TGACGGACTTGGCACAGTAG | | 114 | |
| LXR | CTTCCGCTGGTACTTCCTTTCT | CAGGAGGCCTTCTTCAAGGT | | 70 | |
| GLUT1 | CAAGATGACAGCTCGCCTGAT | AGTCTTCAATCACCTTCTGCGG | | 110 | |
| GLUT12 | TATTGCTGAGATCGCCCCAC | CCTGCAAGGCACCTAATGGA | | 170 | |
| PKM2 | TACGCCACAGTGTACACCAC | CTGTTTGGTCATTGCGGGTG | | 183 | |
| HK2 | TAGACATGGGCTCGCTCAAC | CGCAGATCCGAAGAGAGCTT | | 145 | |
| FASN | GCTAAGATGGCATTGCACGG | TCCATTCAGTTCCAGACGGC | | 135 | |
| SREBP | CAGGGAGAGGGGTGCTTTTC | CAGGCCAGAGCTGATGGAG | | 108 | |
| ACACA | AGTACGTTCGAAGGGCGTACA | ATTCTGTTTGGGTGGGAGGTG | | 120 | |
| CPT1A | GCTCACTACCGAGACATGGG | GACCGGACGGTTTCAGTTCT | | 92 | |
| GPAT | TGGACGTGCCCCATGTGAT | TGCCTCGGATGACTCTCCAT | | 101 | |
| FABP6 | TGAGGGTGATAGTGAGCTCGT | TCCCTCCACCTGCTTTTCATC | | 109 | |
| FBP1 | TAATCTTGTGGCAGCGGGTT | TTCTCCGATTGCCGGATCAA | | 109 | |
| SGLT1 | AGGTTTGCACGAGCCAGATT | TGTGGACACCAAATGCTTCA | | 175 | |
| ZO-1 | CAGATGGCCATAGGGGTGAC | TGGAGTTACCCACAGCTTCC | | 75 | |
| Occludin | AGGTCTGCAACAGCATCACA | ATGCCTTCCCAAAAAGCCCT | | 157 | |
| MUC2 | CCAGACTGGACTTCACGGAC | ACAGCCCCCTCTACCATCAT | | 129 | |

Abbreviations: β-actin: Beta-actin; CYP7B1: Cytochrome P450 family 7 subfamily B member 1; APOB: Apolipoprotein B; LDLR: Low-density lipoprotein receptor; LPL: Lipoprotein lipase; INSR: Insulin receptor; AKT1: AKT serine/threonine kinase 1; PIK3R: Phosphoinositide-3-kinase regulatory subunit; FOXO1: Forkhead box O1; IGF2: Insulin-like growth factor 2; LXR: Liver X receptor; GLUT1: Glucose transporter 1 (facilitated glucose transporter member 1, SLC2A1); GLUT12: Glucose transporter 12 (facilitated glucose transporter member 12, SLC2A12); PKM2: Pyruvate kinase M2; HK2: Hexokinase 2; FASN: Fatty acid synthase; SREBP: Sterol regulatory element-binding protein; ACACA: Acetyl-CoA carboxylase alpha; CPT1A: Carnitine palmitoyltransferase 1A; GPAT: Glycerol-3-phosphate acyltransferase; FABP6: Fatty acid-binding protein 6 (ileal bile acid-binding protein); FBP1: Fructose-1,6-bisphosphatase 1; SGLT1: Sodium-glucose cotransporter 1 (solute carrier family 5 member 1, SLC5A1); ZO-1: Zonula occludens-1 (tight junction protein 1, TJP1); Occludin: Occludin; MUC2: Mucin 2.

Table S2 List of Abbreviations

| Abbreviation | Full Name |
| --- | --- |
| AKT1 | Serine/threonine Kinase 1 |
| CAT | Catalase |
| CPT1A | Carnitine Palmitoyl transferase 1A |
| CYP7B1 | Cytochrome P450 Family 7 Subfamily B1 |
| FABP6 | Fatty Acid Binding Protein 6 |
| FASN | Fatty Acid Synthase |
| FBP1 | Fructose-1,6-Bisphosphatase 1 |
| FCR | Feed Conversion Ratio |
| FOXO1 | Forkhead Box Protein O1 |
| GLUT1 | Glucose Transporter 1 |
| GSH-Px | Glutathione Peroxidase |
| H₂O₂ | Hydrogen Peroxide |
| IGF2 | Insulin-Like Growth Factor 2 |
| IL-1β | Interleukin-1β |
| IL-6 | Interleukin-6 |
| INSR | Insulin Receptor |
| LDLR | Low-Density Lipoprotein Receptor |
| LXR | Liver X Receptor |
| MDA | Malondialdehyde |
| OTUs | Operational Taxonomic Units |
| PKM2 | Pyruvate Kinase M2 |
| SGLT1 | Sodium-Glucose Cotransporter 1 |
| SOD | Superoxide Dismutase |
| SREBP 1/2 | Sterol Regulatory Element-Binding Protein 1/2 |
| T-AOC | Total Antioxidant Capacity |
| TNF-α | Tumor necrosis factor-α |

| Table S3 Standard curve parameters and amplification efficiencies of target genes and reference gene | | | |
| --- | --- | --- | --- |
| Gene | Standard curve equation | R² | Amplification efficiency (%) |
| β-actin | y = -3.259x + 29.861 | 0.9932 | 102.69 |
| CYP7B1 | y = -3.2x + 33.021 | 0.994 | 105.35 |
| APOB | y = -3.228x + 30.006 | 0.997 | 104.07 |
| LDLR | y = -3.341x + 33.843 | 0.9822 | 99.21 |
| LPL | y = -3.484x + 33.241 | 0.9917 | 93.65 |
| INSR | y = -3.1223x + 33.058 | 0.9916 | 109.06 |
| AKT1 | y = -3.1277x + 33.363 | 0.9838 | 108.80 |
| PIK3R | y = -3.198x + 31.475 | 0.991 | 105.44 |
| FOXO1 | y = -3.1353x + 34.471 | 0.9995 | 108.42 |
| IGF2 | y = -3.0953x + 33.091 | 0.9882 | 110.41 |
| LXR | y = -3.178x + 33.381 | 0.9867 | 106.38 |
| GLUT1 | y = -3.2027x + 34.475 | 0.9944 | 105.23 |
| GLUT12 | y = -3.2213x + 33.616 | 0.9935 | 104.38 |
| PKM2 | y = -3.1147x + 31.907 | 0.9878 | 109.44 |
| HK2 | y = -3.1127x + 33.429 | 0.9772 | 109.54 |
| FASN | y = -3.48x + 29.239 | 0.9885 | 93.80 |
| SREBP | y = -3.1523x + 33.788 | 0.9949 | 107.60 |
| ACACA | y = -3.4033x + 31.349 | 0.9943 | 96.71 |
| CPT1A | y = -3.5183x + 32.173 | 0.9908 | 92.41 |
| FABP6 | y = -3.1467x + 31.623 | 0.9995 | 107.87 |
| FBP1 | y = -3.1803x + 30.394 | 0.9931 | 106.27 |
| ZO-1 | y = -3.1703x + 30.201 | 0.9934 | 106.74 |
| Occludin | y = -3.2177x + 29.292 | 0.9982 | 104.54 |
| MUC2 | y = -3.3903x + 30.075 | 0.986 | 97.22 |

Notes: Template cDNA was serially diluted at a 10-fold gradient to generate the standard samples for qPCR. Standard curve equation: Ct = k × log _10_ (template concentration) + b. Amplification efficiency was calculated using the formula E = (10 ^−1/k^ −1) × 100%.

Figure S1. Spearman correlation analysis between organ indices and serum inflammatory and antioxidant parameters in broilers.

Note: The correlation coefficients were calculated using Spearman’s rank correlation analysis. Positive correlations are shown in red, whereas negative correlations are shown in green. Asterisks indicate statistically significant correlations (**P* < 0.05, ***P* < 0.01, **P* < 0.001).

Figure S2. Specificity validation of primers for reference gene.
